# *Besnoitia besnoiti* bradyzoite stages induce suicidal- and rapid vital-NETosis

**DOI:** 10.1017/S0031182019001707

**Published:** 2020-04

**Authors:** Ershun Zhou, Liliana M. R. Silva, Iván Conejeros, Zahady D. Velásquez, Manuela Hirz, Ulrich Gärtner, Philippe Jacquiet, Anja Taubert, Carlos Hermosilla

**Affiliations:** 1 Institute of Parasitology, Justus Liebig University Giessen, Giessen, Germany; 2 Institute of Veterinary Pathology, Justus Liebig University Giessen, Giessen, Germany; 3 Institute of Cell Biology and Anatomy, Justus Liebig University Giessen, Germany; 4 Département Élevage et Produits–Santé Publique Vétérinaire, Laboratoire de Parasitologie et Maladies Parasitaires, École Nationale Vétérinaire de Toulouse (ENVT), Université de Toulouse, Toulouse, France; 5 Département Santé Animale, Interactions Hôtes–Agents Pathogènes (IHAP), Institut National de la Recherche Agronomique (INRA), ENVT, Université de Toulouse, Toulouse, France

**Keywords:** Autophagy, *Besnoitia besnoiti*, bovine PMN, bradyzoites, NETosis, vital NETosis

## Abstract

*Besnoitia besnoiti* is an obligate intracellular apicomplexan protozoan parasite, which causes bovine besnoitiosis. Recently increased emergence within Europe was responsible for significant economic losses in the cattle industry due to the significant reduction of productivity. However, still limited knowledge exists on interactions between *B. besnoiti* and host innate immune system. Here, *B. besnoiti* bradyzoites were successfully isolated from tissue cysts located in skin biopsies of a naturally infected animal, and we aimed to investigate for the first time reactions of polymorphonuclear neutrophils (PMN) exposed to these vital bradyzoites. Freshly isolated bovine PMN were confronted to *B. besnoiti* bradyzoites. Scanning electron microscopy (s.e.m.)- and immunofluorescence microscopy-analyses demonstrated fine extracellular networks released by exposed bovine PMN resembling suicidal NETosis. Classical NETosis components were confirmed *via* co-localization of extracellular DNA decorated with histone 3 (H3) and neutrophil elastase (NE). Live cell imaging by 3D holotomographic microscopy (Nanolive^®^) unveiled rapid vital NETosis against this parasite. A significant increase of autophagosomes visualized by specific-LC3B antibodies and confocal microscopy was observed in *B. besnoiti*-stimulated bovine PMN when compared to non-stimulated group. As such, a significant positive correlation (r = 0.37; *P* = 0.042) was found between *B. besnoiti*-triggered suicidal NETosis and autophagy. These findings suggest that vital- as well as suicidal-NETosis might play a role in early innate host defence mechanisms against released *B. besnoiti* bradyzoites from tissue cysts, and possibly hampering further parasitic replication. Our data generate first hints on autophagy being associated with *B. besnoiti* bradyzoite-induced suicidal NETosis and highlighting for first time occurrence of parasite-mediated vital NETosis.

## Introduction

*Besnoitia besnoiti*, an obligate intracellular apicomplexan parasite, was firstly described in 1912 (Besnoit and Robin, 1912). Several reports on bovine besnoitiosis (i.e. Portugal (Cortes *et al*., 2006), Spain (Fernández-García *et al*., 2009), France (Jacquiet *et al*., 2010), Germany (Schares *et al*., 2009), Italy (Gollnick *et al*., 2010; Rinaldi *et al*., 2013), Switzerland (Basso *et al*., 2013) and Hungary (Hornok *et al*., 2014)) clearly indicate the spread of this disease within Europe (Álvarez-García *et al*., 2013). Based on the increased number of cattle besnoitiosis cases and its geographic expansion into previous non-endemic countries, the European Food Safety Authority (EFSA) classified bovine besnoitiosis as an emerging disease within EU in 2010 (European Food Safety Authority, 2010). Besides Europe, bovine besnoitiosis is also a vastly endemic disease in the Middle East, Asia, South America (Trujillo and Benavides, 2011; Vogelsang and Gallo, 1941) and Africa (Bigalke and Prozesky, 2014; Cortes *et al*., 2014) causing significant economic losses in cattle industry due to significant reduction of productivity (Jacquiet *et al*., 2010; Maqbool *et al*., 2012).

Typically, bovine besnoitiosis is characterized by an acute and a chronic phase with different clinical signs. In the acute phase, *B. besnoiti*-infected cattle present pyrexia, intensive respiratory disorders, increased heart rates, subcutaneous oedema, anasarca, swollen joints, conjunctivitis, nasal discharge, photophobia, reduced milk yield and orchitis associated with permanent infertility in bulls (Bigalke, 1981; Álvarez-García *et al*., 2013; Cortes *et al*., 2014). During the chronic phase of disease, *B. besnoiti* bradyzoites proliferate slowly within the epidermis, subcutaneous tissues, mucous membranes and/or sclera, and form characteristic cysts within mesenchymal host cells, related to dramatic thickening, hardening, folding, wrinkling of the skin (also termed ‘elephant skin’), alopecia, and gradual deterioration of body condition and weight loss (Pols, 1960). Until now, the complete life cycle of *B*. *besnoiti* is not entirely known and final host species are unidentified carnivores. Nevertheless, direct contact between infected and non-infected animals (e.g. natural mating, naso-pharyngeal route) and insect-mediated transmission through biting flies (i. e. tabanids (*Tabanus* spp.), stable flies (*Stomoxys calcitrans*)) have been suggested as suitable transmission routes (Gollnick *et al*., 2015; Gutiérrez-Expósito *et al*., 2017; Tainchum *et al*., 2018) and of epidemiological relevance (Sharif *et al*., 2019).

So far, very limited knowledge exists on early interactions between circulating polymorphonuclear neutrophils (PMN) of host innate immune system with *B. besnoiti*, although these cells are the first ones to be recruited to infection sites. As such, PMN are the most abundant granulocytes in the blood and being the first line of defence against invading pathogens including parasites (Weissmann *et al*., 1980; Behrendt *et al*., 2010; Villagra-Blanco *et al*., 2017*a*). Upon activation, and in addition to phagocytosis (Behrendt *et al*., 2008) and degranulation (Lacy, 2006), PMN also combat efficiently invading pathogens by releasing neutrophil extracellular traps (NETs) (Fuchs *et al*., 2007; Brinkmann and Zychlinsky, 2012; Brinkmann, 2018). These NETs are composed of nuclear DNA decorated with different histones (H1, H2A/H2B, H3, H4) and various antimicrobial granular effector molecules and commonly released *via* a novel cell death process known as suicidal NETosis. Suicidal NETosis is characterized by nuclear and cell membrane rupture and the loss of main PMN functions such as chemotaxis, degranulation and phagocytosis (Fuchs *et al*., 2007; Remijsen *et al*., 2011*b*; Yipp and Kubes, 2013). In contrast, vital NETosis can also occur by not affecting the continuation of mentioned PMN functions (Yipp and Kubes, 2013). Vital NETosis have been demonstrated in response to bacteria (Pilsczek *et al*., 2010), fungi (Byrd *et al*., 2013), LPS-activated platelets (Clark *et al*., 2007) and even the protozoan parasite *Leishmania amazonensis* (Rochael *et al*., 2015). A landmark of vital NETosis is its rapid induction, normally within 30 min after PMN stimulation (Yipp and Kubes, 2013) or as early as 10 min after neutrophil-*L. amazonensis* interaction (Rochael *et al*., 2015). In previous studies, it was shown that suicidal NETosis was able to efficiently trap *B. besnoiti* tachyzoites *in vitro* and that released suicidal NETosis was capable of hampering tachyzoites from active host cell invasion (Muñoz Caro *et al*., 2014*a*). Furthermore, also bovine monocyte-derived extraellular traps (METosis) occurred when these phagocytes have been exposed to vital and motile *B. besnoiti* tachyzoites (Muñoz Caro *et al*., 2014*a*), thereby expanding the spectrum of leukocytes undergoing ETosis (Villagra-Blanco *et al*., 2019).

Conversely, no data are available so far neither on interactions of bovine PMN with *B. besnoiti* bradyzoites nor the role of autophagy in parasite-induced NETosis. Autophagy has recently been indicated to play a crucial role not only influencing classical PMN-mediated effector mechanisms (e.g. phagocytosis) (Mitroulis *et al*., 2010; Skendros *et al*., 2018) but also actively regulating NETosis (Skendros *et al*., 2018). Thus, in the present study, we intended firstly to investigate rapid vital- as well as suicidal-NETosis in bovine PMN exposed to freshly isolated bradyzoites of *B. besnoiti* from subdermal tissue cysts and further to analyse the possible correlation of autophagy in *B. besnoiti* bradyzoite-mediated NETosis.

## Materials and methods

### Animal data

In early 2018, a 4-year-old Limousine heifer (502 kg BW) from South France presented inappetence, limb oedema with desquamation and emaciation. Natural *B. besnoiti* infection was confirmed by polymerase chain reaction investigation. For animal treatment, flunixine meglumine (2.2 mg/kg; Finadyne^®^) and sulfamethoxine (40 mg/kg; Sulfaron^®^) were given. Three months later, the same animal was admitted to the Ecole Nationale Veterinaire Toulouse (ENVT) because of weakness, hyperkeratosis, and multifocal alopecia, and presence of multiple visible cysts within the sclera. One week later, the animal was euthanized due to severe emaciation. At necropsy, bovine besnoitiosis in the scleroderma phase was confirmed: multiple whitish punctuated cysts were observed in sclera and in mucocutaneous junctions of mouth and anus. Moreover, the skin of neck, shoulders, base of tail, hocks, and pasterns presented marked hyperkeratosis with crusty appearance. No other relevant clinical alterations were further noticed. Skin samples of affected areas have been collected, stored in sterile saline solution (4°C) and later on processed for histopathological evaluation as well as for parasite isolation.

### Isolation of vital Besnoitia besnoiti bradyzoites

Skin biopsies were placed in a sterile Petri dish (Nunc) containing a small volume of sterile RPMI 1640 cell culture medium without phenol red (Sigma-Aldrich) supplemented with 1% penicillin-streptomycin (Sigma-Aldrich). A sterile tweezer was used to hold the skin and with a sterile scalpel, the skin surface was carefully scraped in order to release vital bradyzoites from these cysts. As soon as the RPMI 1640 cell culture medium became turbid, it was collected and filtered through a sterile gauze swab in a sieve into a 50-mL Falcon tube followed by centrifugation at 200 × ***g*** for 1 min at room temperature (RT). Then the supernatant was collected and transferred into a new 50-mL Falcon tube, centrifuged (400 × ***g***, 12 min), and the pellet was washed again with RPMI 1640 medium to collect released bradyzoites. All supernatants were collected and centrifuged at 1500 × ***g*** for 10 min, and then the supernatant was discarded and the pellet containing bradyzoites was resuspended in sterile RPMI 1640 cell medium. Vital and extremely motile *B. besnoiti* bradyzoites were isolated and afterwards counted in a Neubauer haemocytometer chamber (Supplementary data video 1). Isolated *B. besnoiti* bradyzoites were firstly stored for 30 min at 4°C and afterwards at −80°C in RPMI 1640 cell medium supplemented with 10% DMSO (Merck).

### Histopathological examination

After successful bradyzoites isolation, parts of skin samples (5 × 5 mm^2^) were stored in 10% phosphate-buffered formalin for histopathological examinations. Shortly, formalin-fixed samples were dehydrated using an ascending ethanol series, embedded in paraffin wax at 56°C and finally sectioned at 3 *μ*m tissue samples at the Institute of Veterinary Pathology, Faculty of Veterinary Medicine, Justus Liebig University Giessen, Germany. Histological tissue samples have been stained using haematoxylin and eosin (HE), periodic acid-Schiff (PAS) and Giemsa staining according to routine protocols and pathological findings/changes of *B. besnoiti*-infected skin samples were then evaluated under a light microscope (Nikon Eclipse 80i) equipped with a DS-Fi1 digital camera (Nikon).

### Isolation of bovine PMN

Healthy adult dairy cows (*n* *=* 3) were served as blood donors. Animals were bled by puncture of the jugular vein and 30 ml peripheral blood was collected in 12 ml heparinized sterile plastic tubes (Kabe Labortechnik). Approximately 20 ml of heparinized blood was re-suspended in 20 ml sterile PBS with 0.02% EDTA (Sigma-Aldrich), slowly layered on top of 12 ml Biocoll^®^ separating solution (density = 1.077 g/L; Biochrom AG), and centrifuged (800 × ***g***, 45 min). After extraction of plasma and peripheral mononuclear blood cells (PBMC), the pellet was washed in 25 ml distilled water and gently shaken during 40 s in order to lyse erythrocytes. Osmolarity was rapidly restored by Hank's balanced salt solution (4 ml, HBSS 10×; Biochrom AG). To complete erythrocyte lysis, this step was repeated twice and PMN were later re-suspended in sterile RPMI 1640 medium (Gibco). Finally, freshly isolated bovine PMN were allowed to rest at 37°C and 5% CO_2_ atmosphere for 30 min until further use (Behrendt *et al*., 2010).

For each experiment, purity and viability of neutrophils were determined. Only samples with a purity of neutrophils higher than 93% and viability greater than 96% (tested by trypan blue exclusion assay (Sigma-Aldrich)) were used.

### Scanning electron microscopy (s.e.m.) analysis

Bovine PMN were co-cultured with vital *B. besnoiti* bradyzoites (ratio 1:4) for 3 h on coverslips (10 mm diameter; Nunc) pre-coated with 0.01% poly-_L_-lysine (Sigma-Aldrich) in an incubator at 37°C and 5% CO_2_ atmosphere. After incubation, cells were fixed in 2.5% glutaraldehyde (Merck), post-fixed in 1% osmium tetroxide (Merck), washed in distilled water, dehydrated, critical point dried by CO_2_-treatment and sputtered with gold. Finally, all samples were visualized *via* a Philips^®^ XL30 scanning electron microscope at the Institute of Anatomy and Cell Biology, Justus Liebig University Giessen, Germany.

### Immunofluorescence microscopy analysis for visualization of B. besnoiti bradyzoite-triggered NETosis

Freshly isolated bovine PMN were co-cultured on 0.01% poly-_L_-lysine pre-treated coverslips (15 mm diameter) with *B. besnoiti* bradyzoites (ratio 1:4) for 3 h (37°C and 5% CO_2_ atmosphere), then fixed by adding 4% paraformaldehyde (Merck) for 15 min and stored at 4°C until further epifluorescence microscopy experiments.

For visualization of suicidal NETosis-related structures, Sytox Orange^®^ (Life Technologies) was used to stain extracellular DNA, anti-histone 3 (H3; clone H11-4, 1:1000; Merck Millipore) and anti-neutrophil elastase (NE) antibodies (AB68672, 1:1000, Abcam) were used to label H3 and NE on NETosis structures. In brief, fixed samples were washed thrice with sterile PBS, then blocked with 1% bovine serum albumin (BSA; Sigma-Aldrich) at RT for 15 min, incubated with corresponding primary antibodies (1 h; RT), and then incubated with secondary antibodies (Alexa Fluor 488 goat anti-mouse IgG or Alexa Fluor 405 goat anti-rabbit IgG, both Life Technologies, 60 min, 1:1000, RT), and finally incubated for 15 min with Sytox Orange^®^ (Life Technologies). After incubation, samples were carefully mounted with the anti-fading solution (ProLong Gold^®^ anti-fading buffer; Thermo Fisher Scientific) and thereafter visualized using an inverted IX81^®^ epifluorescence microscope (Olympus) equipped with a digital camera XM10^®^ (Olympus).

### Live cell interactions between bovine PMN and B. besnoiti bradyzoites investigated by live cell 3d holotomographic microscopy

Isolated PMN (1 × 10^6^) were centrifuged at 300 × ***g*** for 10 min at RT, the supernatant was carefully discarded and cells were suspended in 2 ml of pre-warmed RPMI 1640 cell medium. One ml of this PMN solution was placed in an Ibidi^®^ cell plate 35 mm low profile, and the plate was incubated in an Ibidi^®^ chamber at 5% CO_2_ and 37°C. PMN were allowed to settle down (30 min) to bottom of the plate and then 2 × 10^6^ *B. besnoiti* bradyzoites were added to the center of the plate. The acquisition was set for refractive index (RI; 3D tomography) for a time-lapse of 155 min every 30 s in a Nanolive Fluo-3D Cell Explorer^®^ (Nanolive) microscope. At the end of the experiment, images were exported using Steve software v.2.6^®^ (Nanolive). Using Image J software (Fiji version 1.7, NIH), every frame was exported using z-projection, maximum intensity algorithm and the video was constructed using 1 frame per 5 s of speed. For zoomed video, the region of interest was cropped and the same procedure described above was applied. Digital staining and 3D rendering of activated PMN was performed by using Steve software v.2.6^®^ (Nanolive).

### Autophagosome detection by immunofluorescence analysis

LC3 has been used as a classical marker for autophagosomes (Karim *et al*., 2007), being LC3-I cytosolic and LC3-II membrane bound and enriched in the autophagic vacuole. Therefore, we tested whether LC3 expression might be present during *B. besnoiti* bradyzoite-induced NETosis as described elsewhere (Zhou *et al*., 2019). Briefly, bovine PMN (*n* = 3) were added on 0.01% poly-_L_-lysine pre-coated coverslips (15 mm diameter; Nunc), then stimulated by *B. besnoiti* bradyzoites for 1 h at RT. After incubation, cells were fixed with 4% paraformaldehyde for 10 min, permeabilized with cold methanol (Merck) for 3 min and blocked by using the following blocking buffer (5% BSA (Sigma-Aldtich), 0.1% Triton X-100 (Sigma-Aldrich) in sterile PBS) for 60 min at RT. After removing blocking buffer, cells were incubated overnight at 4°C with rabbit anti-LC3B antibodies (Cat#2775, 1:200, Cell Signaling Technology) diluted in blocking buffer, washed three times with PBS, incubated with goat anti-rabbit IgG conjugated with Alexa Fluor 488 (Invitrogen) for 1 h in the dark at RT. After being washed three times with PBS, coverslips were mounted by prolonged anti-fading reagent with DAPI (Invitrogen) on glass slides (Nunc), and five randomly images were taken per condition using an inverted epifluorescence microscope IX 81^®^ (Olympus) and/or by using confocal microscopy analysis (LSM 710^®^; Zeiss).

### Statistical analysis

Results are illustrated as means ± s.e.m. of at least three independent experimental settings. One-way analysis of variance and Dunnett's multiple comparison test and Spearman correlation test were performed here by using GraphPad Prism 7^®^. Differences were considered significant at a level of *P* ⩽ 0.05.

## Results

### Histopathological examination of B. besnoiti-infected skin

**Fig. 1.**
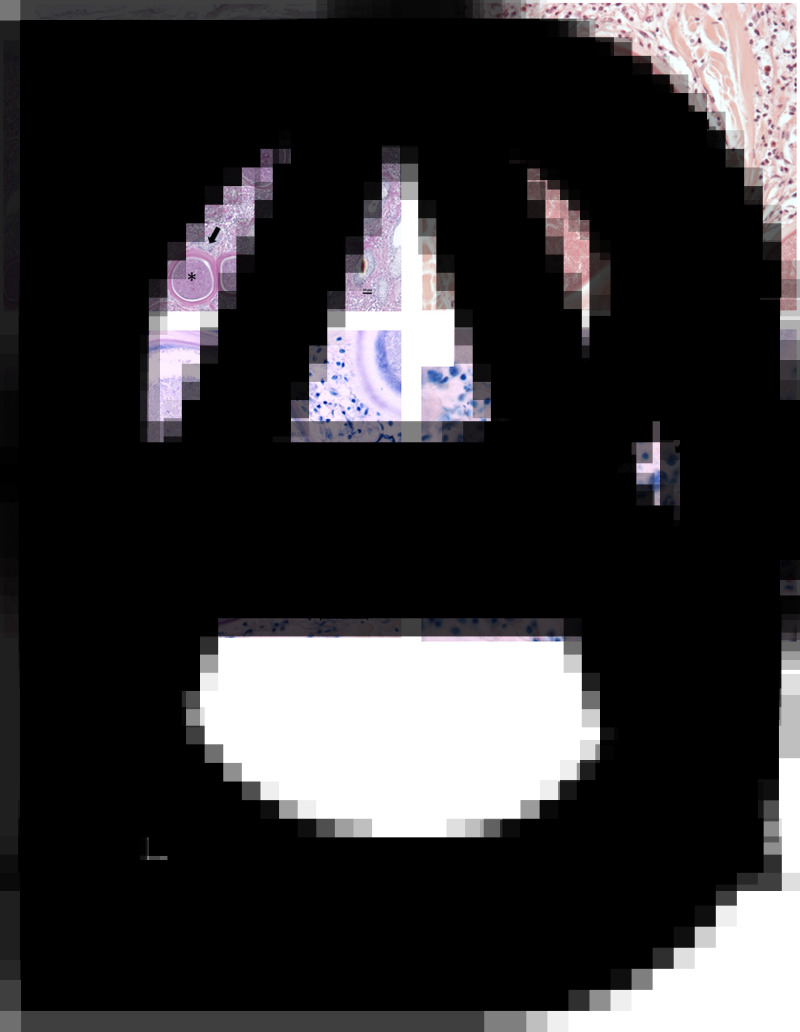
Histopathological examination of skin biopsy (scale bars = 50 *μ*m). (A) Characteristic mature cysts of *Besnoitia besnoiti* within the dermis, Periodic acid–Schiff (PAS) staining, 40 × total magnification; (B) A mature cyst of *Besnoitia besnoiti* with a three-layered wall composed of an outer (black arrow), middle (clear arrow) and inner wall (arrowhead), haematoxylin and eosin (H&E) staining, 200 × total magnification; (C) A mature cyst of *Besnoitia besnoiti* with a three-layered wall composed of an outer (black arrow), middle (clear arrow) and inner wall (arrowhead), Giemsa staining, 200 × total magnification; (D) Vicinity of a mature *Besnoitia besnoiti* cyst with an inflammatory infiltrate composed of macrophages (black arrow), fewer lymphocytes and plasma cells (black arrowhead), neutrophils (red arrowhead) and eosinophils (clear arrow) as well as rare multinucleated giant cells (asterisk), Giemsa staining, 400 ×  total magnification. Scale bar = 50 *μ*m.

Histopathological examination revealed multifocal large-sized round to ovoid *B. besnoiti*-cysts present in the dermis, panniculus and underlying muscle layer (Fig. 1A, highlighted exemplary by black arrows). Rare early tissue cysts were small, approximately 10–20 *μ*m and contained a parasitophorous vacuole (PV) with few banana-shaped 3–5 *μ*m structures (bradyzoites). Mature *B. besnoti*-cysts (up to 400 *μ*m) were filled with thousands of bradyzoite stages (Fig. 1A-C, asterisks). Cysts containing numerous typical banana-shaped *B. besnoiti* bradyzoites had a three-layered wall (Fig. 1B and C, thickness of 10–30 *μ*m thick): the outer wall composed of compressed collagen type I fibres (Fig. 1B and C, black arrows), the middle layer representing a thick hyaline capsule composed of extracellular matrix (Fig. 1B and C, clear arrows) and the inner layer composed of a small rim of host cell cytoplasm with often multiple flattened nuclei containing the PV (Fig. 1B and C, arrowheads). The outer wall of compressed collagen, as well as the bradyzoites, stained mildly and the middle hyaline layer were brightly stained with PAS (Fig. 1A). Using Giemsa stain, the inner rim of the middle hyaline layer of mature cysts stained purple, while the outer rim of the hyaline layer was translucent (Fig. 1C, clear arrow) and the inner layer containing the host cell cytoplasm as well as the bradyzoites themselves stained blue (Fig. 1C, arrowhead and asterisk). Surrounding the tissue cysts there was a mild to moderate multifocal to coalescing infiltrate (Fig. 1D) composed of macrophages (Fig. 1D, black arrow), fewer lymphocytes, plasma cells (Fig. 1D, black arrowhead), neutrophils (Fig. 1D, red arrowhead) and eosinophils (Fig. 1D, clear arrow) and rare multinucleated giant cells (Fig. 1D, asterisk). Few tissue cysts were found ruptured and surrounded by abundant macrophages including multinucleated giant cells, numerous neutrophils and eosinophils, which were sometimes arranged in clusters as well as fewer lymphocytes and plasma cells. Also, throughout the affected skin, there was mild to moderate diffuse epithelial hyperplasia and moderate orthokeratotic hyperkeratosis.

### Besnoitia besnoiti bradyzoite-triggered NETosis was unveiled via s.e.m.- and immunofluorescence microscopy-analysis

**Fig. 2.**
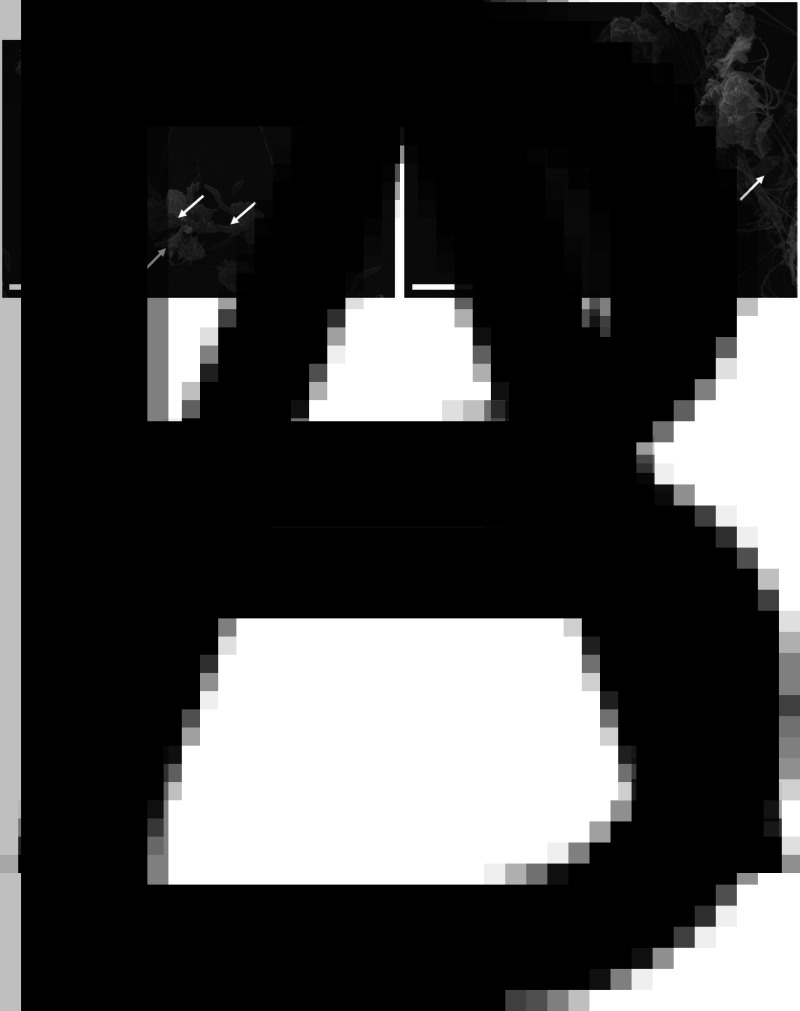
NETosis of bovine PMN after a confrontation with *Besnoitia besnoiti* bradyzoites. Scanning electron microscopy (s.e.m.) analysis revealed NETosis being formed by bovine PMN co-cultured with *B. besnoiti* bradyzoites, and these extracellular structures resulted in a fine meshwork containing bardyzoites as indicated by white arrows. Scale bar = 5 *μ*m.

To investigate whether *B. besnoiti* bradyzoites were capable to induce NETosis, bovine PMN exposed to bradyzoites were analysed by s.e.m. Fine network structures were observed in *B. besnoiti* bradyzoites-stimulated bovine PMN (Fig. 2), and many bradyzoites were trapped by those structures (Fig. 2) visualized in s.e.m. analysis. Alongside, different morphologies of bovine PMN were observed around these fine networks. Whilst typical smooth rounded PMN have been found in close proximity to bradyzoites indicating a rather inactivation status, other exposed PMN showed disrupted cell membrane surfaces and thereby releasing extracellular filaments entrapping firmly bradyzoites by cell death. Former PMN status corresponded well to previously described suicidal (lytic) ETosis against this apicomplexan protozoa where extracellular fibres mainly derived from dead PMN and monocytes (Muñoz-Caro *et al*., 2014*a*,*b*).

**Fig. 3.**
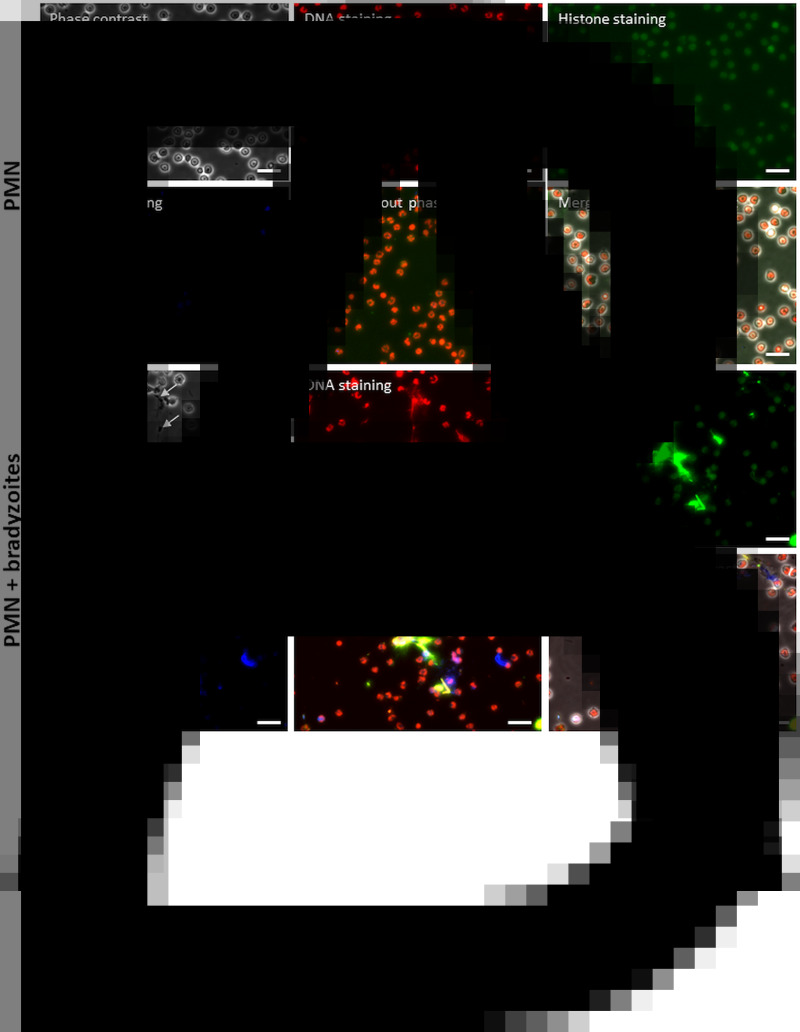
Suicidal NETosis was visualized by co-localization of DNA with histones (H3) and neutrophil elastase (NE) in *B. besnoiti* bradyzoite-exposed bovine PMN. After 3 h of incubation, co-cultures of bovine PMN and *B. besnoiti* bradyzoites in a 1:4 ratio were fixed, permeabilized, and then suicidal NETosis was visualized *via* immunostaining. Panel A: PMN alone group; Panel B: PMN + bradyzoites group. Bradyzoites were indicated by arrows. Scale bar = 20 *μ*m.

In order to confirm whether bovine PMN were undergoing suicidal NETosis, main components of NET formation (i.e. DNA, histones (H3) and NE) were visualized *via* immunostaining. In the control group (non-exposed PMN), no NETosis-like structures were observed by co-localization of H3 and NE (Fig. 3A). In contrast, the classical characteristics of suicidal NETosis were demonstrated in bovine PMN exposed to *B. besnoiti* bradyzoites by co-localization of extracellular DNA adorned with H3 and NE (Fig. 3B), and several bradyzoites being firmly trapped by NETosis as indicated by white arrows in Fig. 3B.

### 3D-holotomographic microscopy live cell imaging of B. besnoiti bradyzoite-triggered vital NETosis

**Fig. 4.**
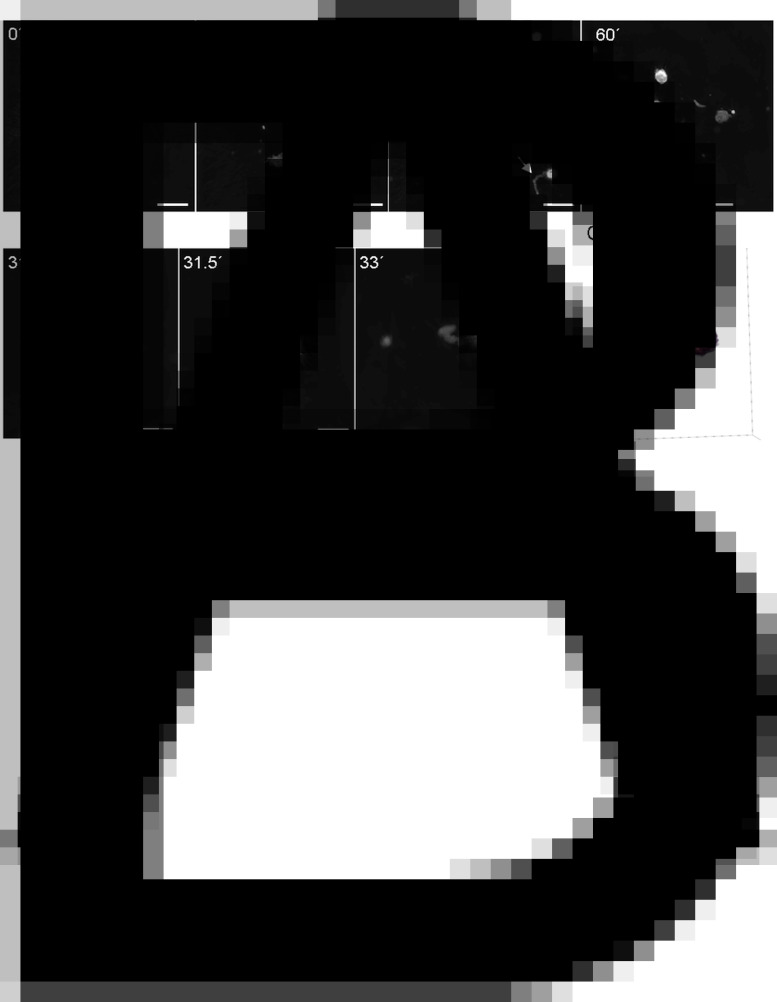
*Besnoitia besnoiti* bradyzoites induced vital NETosis. Live cell 3D holotomographic microscopy (Nanolive^®^) analysis under controlled temperature and atmosphere conditions was performed for 1 h of interactions registering images every 30 s (A). At 31 min of incubation a tossing vital NETosis is observed without compromising the overall structure of PMN (B). Digital staining and 3D holotomographic reconstruction of tossed vital NETosis (C). (A) Scale bar = 20 *μ*m, (B) Scale bar = 10 *μ*m.

Activation of bovine PMN and NETosis were additionally analysed by live cell 3D-holotomographic microscopy technology (Nanolive^®^). Activation of PMN occurred within the first 5 to 30 min of interaction with motile bradyzoites thereby showing pseudopod formation and rapid migration and crawling activities of PMN into the vision field showing bradyzoites. Noteworthy to mention was the observation of an elongated structure being rapidly tossed out from PMN after 30 min of parasite interaction. Due to the time point of occurrence and the non-lytic PMN phenotype of this ‘chameleon tongue-like’ reactions we interpreted this response as vital NETosis (Fig. 4A-B; please refer to Video S2). The digital staining and 3D reconstruction of vital NETosis showed clearly that neither the overall cell phenotype nor crawling activities were altered by the protrusion of this elongated structure (Video S3; Fig. 4C).

### Autophagy occurred during B. besnoiti-triggered suicidal NETosis

**Fig. 5.**
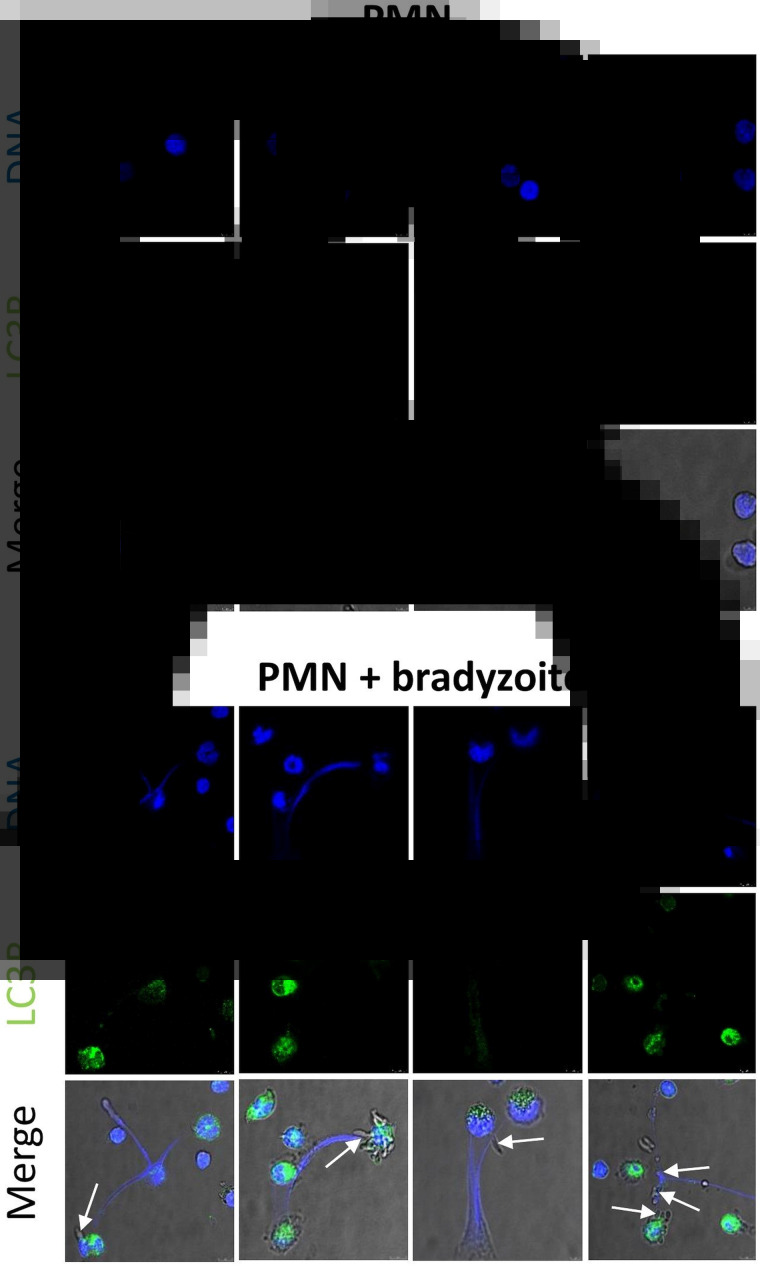
Autophagy occurs in *Besnoitia besnoiti*-triggered suicidal NETosis. Bovine PMN (*n* = 3) were exposed to *B. besnoiti* bradyzoites on coverslips for 1 h at 37°C, 5% CO_2_. Samples were fixed and thereafter permeabilized for LC3B-based immunostaining in order to determine autophagosome formation by confocal microscopy analysis. Bradyzoites were indicated by arrows in merged images. Scale bar = 10 *μ*m.

Autophagy is a highly conserved intracellular degradation process not only to keep homeostasis or energy source of mammalian cells but also pivotal in several host innate immune functions (Germic *et al*., 2019). During autophagy, LC3 (microtubule-associated protein 1A/1B-light chain 3) is an important protein being involved in autophagosome formation, and it has been used as a classical marker of autophagosomes. As shown in Fig. 5, the majority of bovine PMN (see Fig. 5A) were still round and inactive without stimulation of *B. besnoitia* bradyzoites. In contrast, most of bovine PMN exposed to *B. besnoitia* bradyzoites (see Fig. 5B) were undergoing autophagy alongside with NETosis resulting in bradyzoite entrapment (Fig. 5B), indicating an association of these two cellular processes.

**Fig. 6.**
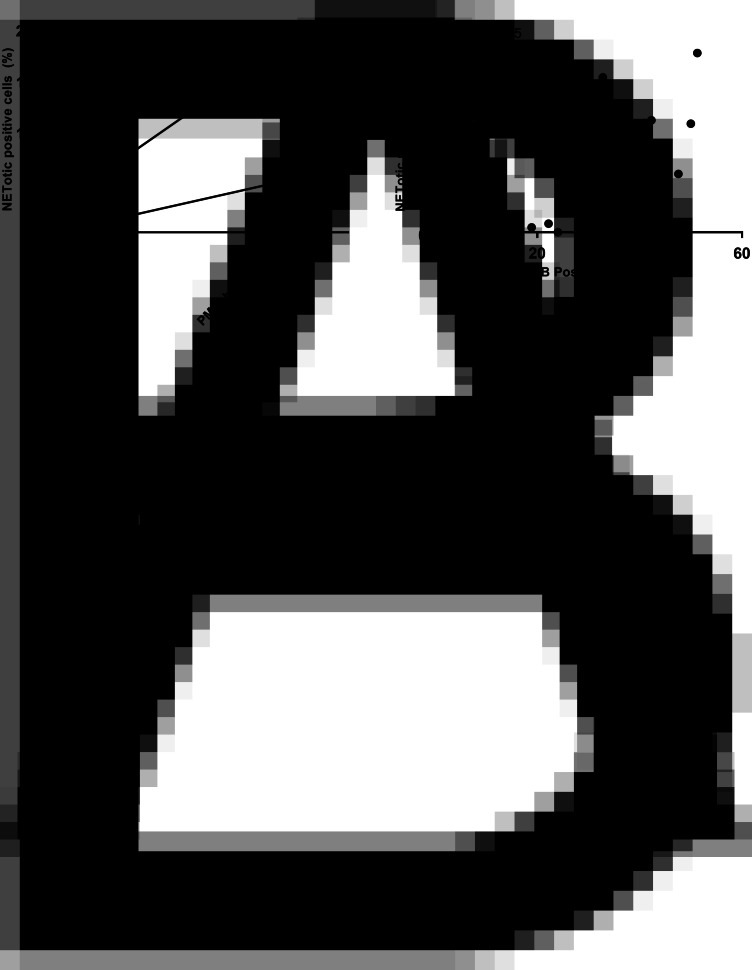
Autophagy has a significant correlation with *Besnoitia besnoiti* bradyzoite-triggered suicidal NETosis. Five images were randomly taken from each sample, the number of NETotic (A) and LC3B-positive PMN were counted using ImageJ and the percentages over total cells was calculated. Positive correlation between *B. besnoiti*-induced LC3B expression and NETotic cells was analysed by Spearman test (B). Results are represented as a before-after graph with data derived from three different animals (*n* = 3). *P* values of <0.05 were considered as statistically significant.

To investigate in more detail concomitant autophagy while *B. besnoiti*-induced NETosis, the percentages of ‘NETotic cells’ and LC3B-positive cells were calculated, and thereafter a Spearman correlation test was performed. As seen in Fig. 6A, more cells were undergoing suicidal NETosis in *B. besnoiti* bradyzoite-stimulated bovine PMN when compared to non-stimulated PMN (not statistically significant). Moreover, a significant low positive correlation (r = 0.3735, *P* = 0.042) was found between suicidal NETosis and autophagy in *B. besnoiti* bradyzoite-stimulated bovine PMN (Fig. 6B) compared to negative controls.

## Discussion

Bovine besnoitiosis is caused by the parasite *B. besnoiti* which is a cyst forming apicomplexan parasite closely related to *Toxoplasma gondii* and *Neospora caninum* (Ellis *et al*., 2000). Chronic bovine besnoitiosis is characterized by tissue cyst formation, especially in the skin and in the mucosa of diverse organs (e.g. eyes, genitals) with associated progressive thickening, folding, hardening, wrinkling lesions of affected skin or mucosa. Characteristic large-sized cysts of *B. besnoiti* containing thousands of bradyzoites were identified in the skin samples of this naturally infected heifer by histopathological examinations in accordance to previous reports (Cortes *et al*., 2005; Jacquiet *et al*., 2010; Rostaher Ana *et al*., 2010; Gentile *et al*., 2012; Frey *et al*., 2013). The affected animal did not benefit from implemented treatments and its deteriorated clinical status determined euthanasia. However, even if the clinical status of this animal had remained unaltered, culling would have been the better control measurement for cattle besnoitiosis in the farm.

NETosis is an effective defence process of activated PMN to ensnare and eliminate invading pathogens by releasing web-like extracellular traps which consist of DNA as a backbone, histones (H1, H2A/H2B, H3, H4), and diverse anti-microbial peptides/proteases such as cathepsin G, *α*-defensin, pentraxin, cathelicidin (LL37), lactoferrin, calprotectin and others (Amulic and Hayes, 2011; Hermosilla *et al*., 2014). Recently, more attention has been paid on pivotal role of NETosis against protozoan- and metazoan-parasites in various terrestrial and marine mammalian species (Behrendt *et al*., 2010; Silva *et al*., 2014; Muñoz Caro *et al*., 2014*a*; Reichel *et al*., 2015; Rochael *et al*., 2015; Villagra-Blanco *et al*., 2017*b*) as well as gastropods (Lange *et al*., 2017). Consequently, PMN-derived NETosis and monocyte-derived METosis exerted potent entrapment capacities against *B. besnoiti* tachyzoites indicating that these two leukocyte populations might reduce parasite replication during the acute phase of infection as previously postulated. Nevertheless, no data in the literature are still available on early NETosis against *B. besnoiti* bradyzoites although bradyzoites are released from tissue cysts *in vivo* (Langenmayer *et al*., 2015).

Here, for the first time, we demonstrated that bradyzoites of *B. besnoiti* were also able to induce NETosis in a similar manner as tachyzoites, and thus proving that *B. besnoiti*-triggered NETosis is a rather parasite stage-independent effector mechanism. Bradyzoites and tachyzoites of *B. besnoiti* are known to exhibit different antigens (Fernandez-Garcia *et al*., 2009; Schares *et al*., 2010), and whether bradyzoite-specific antigens induced bovine NETosis could not be answered here (Silva *et al*., 2014; Muñoz-Caro *et al*., 2015, 2018). In line with these findings, other reports have also shown that different apicomplexan parasitic stages of the same species are able to induce NETosis (Silva *et al*., 2014; Muñoz-Caro *et al*., 2015; Villagra-Blanco *et al*., 2017*b*). s.e.m. analysis unveiled the presence of classical web-like structures released by bovine PMN exposed to *B. besnoiti* bradyzoites, as previously observed for tachyzoites (Muñoz Caro *et al*., 2014*a*; Maksimov *et al*., 2016). Additionally, the main components of NETosis (i.e. DNA, histones (H3), NE) were identified here and visualized *via* immunostaining and proving that these web-like structures were mainly suicidal NETosis. In addition, live cell imaging by 3D holotomographic microscopy showed rapid vital NETosis within the first 30 min without compromising the general structure of PMN cell membrane as well as crawling activity. In the past, it has been proposed that PMN subpopulations are able to elicit different types of NETosis and that only 20–30% undergo suicidal NETosis (Fuchs *et al*., 2007; Yipp and Kubes, 2013). Interestingly, suicidal NETosis seems to be more related to chemical stimuli as PMA, requiring hours to occur, meanwhile, vital NETosis might be more related to biological triggering agents of NETosis such as bacteria, fungi (Yipp and Kubes, 2013) or parasites (Rochael *et al*., 2015). Currently, it is not clear how vital NETosis could contribute to the control of infection, but it has been suggested that this process is the first effector mechanism against invasive pathogens to occur (Yipp and Kubes, 2013) and afterwards followed by suicidal NETosis, which results in stronger DNA release. Additionally, it has been speculated that this phenomenon could be initialized by TRL mediated responses (de Buhr and von Köckritz-Blickwede, 2016), but further investigations are necessary to better understand such an intriguing feature in bovine besnoitiosis. To our knowledge, this is the first time that vital NETosis is evidenced by 3D live cell imaging as a PMN response to motile parasite stages.

As stated above, bradyzoites can be released from tissue cysts either after host induced- or after mechanical rupture (Schulz, 1960; Langenmayer *et al*., 2015). Further, Langenmayer *et al*. (2015) suggested that during chronic bovine besnoitiosis intravascular circulation of ‘zoites’ might be possible after mechanical rupture of cysts located directly underneath vascular endothelium or after reactivation of tissue cysts and stage conversion into tachyzoite stages. Irrespective of these *in vivo* scenarios, released bradyzoites would be immediately in close contact to PMN and extruded NETosis might ultimately hamper bradyzoite dissemination. Released bradyzoites might not be immediately identified in the bloodstream of infected animals due to pro-inflammatory host innate immune reactions as proposed elsewhere (Langenmayer *et al*., 2015) and *in vivo* PMN are among the first ones to be recruited to inflammation/infection sites (Fuchs *et al*., 2007; Villagra-Blanco *et al*., 2019; Zhou *et al*., 2019).

Our present results also demonstrated that autophagy was associated with bradyzoite-triggered NETosis. These findings corresponded well to recent data on *B. besnoiti* tachyzoite-mediated suicidal NETosis with concomitant autophagy (Zhou *et al*., 2019). While autophagy process has recently been tightly associated with major neutrophil functions, including degranulation, reactive oxygen species production, and release of neutrophil extracellular traps, the exact molecular mechanisms and autophagy pathways are still not completely clear (Remijsen *et al*., 2011*a*; Ullah *et al*., 2017; Skendros *et al*., 2018). Also, autophagy is an essential intracellular degradation mechanism to regulate protein and organelle turnover in many living cells thereby maintaining homeostasis and intracellular energy balance (Levine and Kroemer, 2008). During the process of autophagy, intracellular autophagosomes ultimately fuse with lysosomes to degrade and recycle the inside cargo (Bernard and Klionsky, 2013). LC3 is a small soluble protein, which is distributed ubiquitously in mammalian tissues and known to form stable associations with the membrane of autophagosomes (Tanida *et al*., 2008). Thus, LC3 is widely used as a classical marker for microscopical detection of autophagosomes (Koukourakis *et al*., 2015; Park *et al*., 2017). Previous studies have revealed that autophagy is required for NETosis (Remijsen *et al*., 2011*a*; Ullah *et al*., 2017), and that autophagy induction significantly increased NETosis (Park *et al*., 2017). Accordingly, LC3B-stained autophagosomes were detected concomitantly in PMN extruding suicidal NETosis towards *B. besnoiti* bradyzoites. These findings confirm that autophagy is required in bovine NETosis not only against *B. besnoiti* tachyzoite- (Zhou *et al*., 2019) but also against bradyzoite-stages as Spearman test revealed a significant low positive correlation between these two processes. In agreement to our findings, autophagy has also been reported to prime PMN not only for increased NETosis but also for increased phagocytosis during sepsis (Park *et al*., 2017). PMN-derived phagocytosis through autophagy is expected to occur in the chronic phase of cattle besnoitosis *in vivo*, however this process needs further investigations.

In summary, we describe for the first time the ability of bovine PMN to cast NETosis against motile *B. besnoiti* bradyzoites evidencing the importance of this ancient and well-conserved effector mechanism of early host innate immune system in cattle. Furthermore, LC3B-stained autophagosomes were detected in *B. besnoiti* bradyzoite-exposed PMN casting NETs resulting in a significant low positive correlation of autophagy and parasite-induced suicidal NETosis. Nevertheless, further autophagy-related investigations should elucidate other molecular mechanisms in this cell pathway, such as potential membrane changes of mitochondria and oxygen consumption rates, as well as the role of AMPK in autophagy (Zhou *et al*., 2019). Finally, *B. besnoiti*-mediated vital NETosis resulted in a rapid extrusion and retraction of a ‘chameleon tongue-like’ structure, which is the first hint for this type of NETosis against these apicomplexan parasites. Exact machinery, *B. besnoiti*-specific antigens and PMN receptors leading to fast parasite-triggered vital NETosis need further investigations.

## Supplementary material

For supplementary material accompanying this paper visit https://doi.org/10.1017/S0031182019001707. click here to view supplementary material

## References

[ref1] Álvarez-García G, Frey CF, Mora LMO and Schares G (2013) A century of bovine besnoitiosis: an unknown disease re-emerging in Europe. Trends in Parasitology 29, 407–415. 23830145 10.1016/j.pt.2013.06.002

[ref2] Amulic B and Hayes G (2011) Neutrophil extracellular traps. Current Biology 21, R297–R298. 21549944 10.1016/j.cub.2011.03.021

[ref3] Basso W, Lesser M, Grimm F, Hilbe M, Sydler T, Trösch L, Ochs H, Braun U and Deplazes P (2013) *Bovine besnoitiosis* in Switzerland: imported cases and local transmission. Veterinary Parasitology 198, 265–273. 24120579 10.1016/j.vetpar.2013.09.013

[ref4] Behrendt JH, Taubert A, Zahner H and Hermosilla C (2008) Studies on synchronous egress of coccidian parasites (*Neospora caninum*, *Toxoplasma Gondii*, *Eimeria bovis*) from bovine endothelial host cells mediated by calcium ionophore A23187. Veterinary Research Communications 32, 325–332. 18158611 10.1007/s11259-007-9033-7

[ref5] Behrendt JH, Ruiz A, Zahner H, Taubert A and Hermosilla C (2010) Neutrophil extracellular trap formation as innate immune reactions against the apicomplexan parasite *Eimeria bovis*. Veterinary Immunology and Immunopathology 133, 1–8. 19625090 10.1016/j.vetimm.2009.06.012

[ref6] Bernard A and Klionsky DJ (2013) Autophagosome formation: tracing the source. Developmental cell 25, 116–117. 23639440 10.1016/j.devcel.2013.04.004 PMC3668556

[ref7] Besnoit C and Robin V (1912) Sarcosporidiose cutanée chez une vache. Revue Vétérinaire 37, 649–663.

[ref8] Bigalke RD (1981) Besnoitiosis and globidiosis. In Ristic M and McIntyre WIM (eds), Diseases of Cattle in the Tropics. Current Topics in Veterinary Medicine and Animal Science, 6. Dordrecht: Springer, pp. 429–442.

[ref9] Bigalke RD and Prozesky L (2014) Besnotiosis. In Coetzer JAW and Tustin RC (eds), Infectious Diseases of Livestock, vol. 1. Cape Town: Oxford University Press, pp. 331–359.

[ref10] Brinkmann V (2018) Neutrophil extracellular traps in the second decade. Journal of Innate Immunity 10, 414–421. 29909412 10.1159/000489829 PMC6784051

[ref11] Brinkmann V and Zychlinsky A (2012) Neutrophil extracellular traps: is immunity the second function of chromatin? The Journal of Cell Biology 198, 773–783. 22945932 10.1083/jcb.201203170 PMC3432757

[ref12] Byrd AS, O'Brien XM, Johnson CM, Lavigne LM and Reichner JS (2013) An extracellular matrix–based mechanism of rapid neutrophil extracellular trap formation in response to *Candida albicans*. The Journal of Immunology 190, 4136–4148. 23509360 10.4049/jimmunol.1202671 PMC3622194

[ref13] Clark SR, Ma AC, Tavener SA, McDonald B, Goodarzi Z, Kelly MM, Patel KD, Chakrabarti S, McAvoy E and Sinclair GD (2007) Platelet TLR4 activates neutrophil extracellular traps to ensnare bacteria in septic blood. Nature medicine 13, 463. 10.1038/nm1565 17384648

[ref14] Cortes H, Leitao A, Vidal R, Vila-Vicosa MJ, Ferreira ML, Caeiro V and Hjerpe CA (2005) Besnoitiosis in bulls in Portugal. Veterinary Record 157, 262–264. 16127139 10.1136/vr.157.9.262

[ref15] Cortes HCE, Reis Y, Waap H, Vidal R, Soares H, Marques I, Pereira da Fonseca I, Fazendeiro I, Ferreira ML, Caeiro V, Shkap V, Hemphill A and Leitão A (2006) Isolation of *Besnoitia Besnoiti* from infected cattle in Portugal. Veterinary Parasitology 141, 226–233. 16822614 10.1016/j.vetpar.2006.05.022

[ref16] Cortes H, Leitão A, Gottstein B and Hemphill A (2014) A review on bovine besnoitiosis: a disease with economic impact in herd health management, caused by *Besnoitia Besnoiti* (franco and borges,). Parasitology 141, 1406–1417. 24694568 10.1017/S0031182014000262

[ref17] de Buhr N and von Köckritz-Blickwede M (2016) How neutrophil extracellular traps become visible. Journal of Immunology Research 2016, 4604713. 27294157 10.1155/2016/4604713 PMC4884809

[ref18] Ellis JT, Holmdahl OJM, Ryce C, Njenga JM, Harper PAW and Morrison DA (2000) Molecular phylogeny of *Besnoitia* and the genetic relationships among *Besnoitia* of cattle, wildebeest and goats. Protist 151, 329–336. 11212893 10.1078/S1434-4610(04)70031-0

[ref19] European Food Safety Authority (2010) Bovine Besnoitiosis: an emerging disease in Europe. EFSA Journal 8, 1499.

[ref20] Fernandez-Garcia A, Alvarez-Garcia G, Risco-Castillo V, Aguado-Martinez A, Marugan-Hernandez V and Ortega-Mora LM (2009) Pattern of recognition of *Besnoitia Besnoiti* tachyzoite and bradyzoite antigens by naturally infected cattle. Veterinary parasitology 164, 104–110. 19595513 10.1016/j.vetpar.2009.06.020

[ref21] Fernández-García A, Risco-Castillo V, Pedraza-Díaz S, Aguado-Martínez A, Álvarez-García G, Gómez-Bautista M, Collantes-Fernández E and Ortega-Mora LM (2009) First isolation of *Besnoitia Besnoiti* from a chronically infected Cow in Spain. Journal of Parasitology 95, 474–476. 18803440 10.1645/GE-1772.1

[ref22] Frey CF, Gutiérrez-Expósito D, Ortega-Mora LM, Benavides J, Marcén JM, Castillo JA, Casasús I, Sanz A, García-Lunar P, Esteban-Gil A and Álvarez-García G (2013) Chronic bovine besnoitiosis: intra-organ parasite distribution, parasite loads and parasite-associated lesions in subclinical cases. Veterinary Parasitology 197, 95–103. 23680543 10.1016/j.vetpar.2013.04.023

[ref23] Fuchs TA, Abed U, Goosmann C, Hurwitz R, Schulze I, Wahn V, Weinrauch Y, Brinkmann V and Zychlinsky A (2007) Novel cell death program leads to neutrophil extracellular traps. The Journal of Cell Biology 176, 231–241. 17210947 10.1083/jcb.200606027 PMC2063942

[ref24] Gentile A, Militerno G, Schares G, Nanni A, Testoni S, Bassi P and Gollnick NS (2012) Evidence for bovine besnoitiosis being endemic in Italy—first in vitro isolation of *Besnoitia Besnoiti* from cattle born in Italy. Veterinary Parasitology 184, 108–115. 21978744 10.1016/j.vetpar.2011.09.014

[ref25] Germic N, Frangez Z, Yousefi S and Simon H-U (2019) Regulation of the innate immune system by autophagy: neutrophils, eosinophils, mast cells, NK cells. Cell Death & Differentiation 26, 703–714. 30737478 10.1038/s41418-019-0295-8 PMC6460399

[ref26] Gollnick NS, Gentile A and Schares G (2010) Diagnosis of bovine besnoitiosis in a bull born in Italy. The Veterinary record 166, 599–599. 20453244 10.1136/vr.c2314

[ref27] Gollnick NS, Scharr JC, Schares G and Langenmayer MC (2015) Natural *Besnoitia Besnoiti* infections in cattle: chronology of disease progression. BMC veterinary research 11, 35. 25886463 10.1186/s12917-015-0344-6 PMC4357170

[ref28] Gutiérrez-Expósito D, Ferre I, Ortega-Mora LM and Álvarez-García G (2017) Advances in the diagnosis of bovine besnoitiosis: current options and applications for control. International journal for parasitology 47, 737–751. 28893637 10.1016/j.ijpara.2017.08.003

[ref29] Hermosilla C, Caro TM, Silva LMR, Ruiz A and Taubert A (2014) The intriguing host innate immune response: novel anti-parasitic defence by neutrophil extracellular traps. Parasitology 141, 1489–1498. 24721985 10.1017/S0031182014000316

[ref30] Hornok S, Fedák A, Baska F, Hofmann-Lehmann R and Basso W (2014) *Bovine besnoitiosis* emerging in central-Eastern Europe, Hungary. Parasites & Vectors 7, 20. 24410743 10.1186/1756-3305-7-20 PMC3895772

[ref31] Jacquiet P, Liénard E and Franc M (2010) *Bovine besnoitiosis*: epidemiological and clinical aspects. Veterinary Parasitology 174, 30–36. 20850933 10.1016/j.vetpar.2010.08.013

[ref32] Karim MR, Kanazawa T, Daigaku Y, Fujimura S, Miotto G and Kadowaki M (2007) Cytosolic LC3 ratio as a sensitive Index of macroautophagy in isolated Rat hepatocytes and H4-II-E cells. Autophagy 3, 553–560. 17617739 10.4161/auto.4615

[ref33] Koukourakis MI, Kalamida D, Giatromanolaki A, Zois CE, Sivridis E, Pouliliou S, Mitrakas A, Gatter KC and Harris AL (2015) Autophagosome proteins LC3A, LC3B and LC3C have distinct subcellular distribution kinetics and expression in cancer cell lines. PLOS ONE 10, e0137675. 26378792 10.1371/journal.pone.0137675 PMC4574774

[ref34] Lacy P (2006) Mechanisms of degranulation in neutrophils. Allergy, Asthma, and Clinical Immunology: Official Journal of the Canadian Society of Allergy and Clinical Immunology 2, 98–108. 20525154 10.1186/1710-1492-2-3-98 PMC2876182

[ref35] Lange MK, Penagos-Tabares F, Muñoz-Caro T, Gärtner U, Mejer H, Schaper R, Hermosilla C and Taubert A (2017) Gastropod-derived haemocyte extracellular traps entrap metastrongyloid larval stages of *Angiostrongylus Vasorum*, *Aelurostrongylus abstrusus* and *Troglostrongylus brevior*. Parasites & vectors 10, 50. 28143510 10.1186/s13071-016-1961-z PMC5282800

[ref36] Langenmayer MC, Gollnick NS, Majzoub-Altweck M, Scharr JC, Schares G and Hermanns W (2015) Naturally acquired *Bovine Besnoitiosis*: histological and immunohistochemical findings in acute, subacute, and chronic disease. Veterinary Pathology 52, 476–488. 25096291 10.1177/0300985814541705

[ref37] Levine B and Kroemer G (2008) Autophagy in the pathogenesis of disease. Cell 132, 27–42. 18191218 10.1016/j.cell.2007.12.018 PMC2696814

[ref38] Maksimov P, Hermosilla C, Kleinertz S, Hirzmann J and Taubert A (2016) *Besnoitia Besnoiti* infections activate primary bovine endothelial cells and promote PMN adhesion and NET formation under physiological flow condition. Parasitology Research 115, 1991–2001. 26847631 10.1007/s00436-016-4941-5

[ref39] Maqbool MS, Bhat SA, Shah SN, Ganayi BA and Sheikh TA (2012) Bovine Besnoitiosis-impact on profitable cattle production. International Journal of Livestock Research 2, 78–81.

[ref40] Mitroulis I, Kourtzelis I, Kambas K, Rafail S, Chrysanthopoulou A, Speletas M and Ritis K (2010) Regulation of the autophagic machinery in human neutrophils. European journal of immunology 40, 1461–1472. 20162553 10.1002/eji.200940025

[ref41] Muñoz Caro T, Hermosilla C, Silva LMR, Cortes H and Taubert A (2014a) Neutrophil extracellular traps as innate immune reaction against the emerging apicomplexan parasite *Besnoitia besnoiti*. PLoS ONE 9, e91415. 24618849 10.1371/journal.pone.0091415 PMC3950022

[ref42] Muñoz-Caro T, Silva LM, Ritter C, Taubert A and Hermosilla C (2014b) *Besnoitia Besnoiti* tachyzoites induce monocyte extracellular trap formation. Parasitology research 113, 4189–4197. 25193048 10.1007/s00436-014-4094-3

[ref43] Muñoz-Caro T, Mena Huertas SJ, Conejeros I, Alarcón P, Hidalgo MA, Burgos RA, Hermosilla C and Taubert A (2015a) *Eimeria bovis*-triggered neutrophil extracellular trap formation is CD11b-, ERK 1/2–, p38 MAP kinase- and SOCE-dependent. Veterinary Research 46, 23. 25885264 10.1186/s13567-015-0155-6 PMC4349228

[ref44] Muñoz-Caro T, Conejeros I, Zhou E, Pikhovych A, Gärtner U, Hermosilla C, Kulke D and Taubert A (2018) *Dirofilaria immitis* microfilariae and third-stage Larvae induce canine NETosis resulting in different types of neutrophil extracellular traps. Frontiers in Immunology 9, 968. 29867950 10.3389/fimmu.2018.00968 PMC5951940

[ref45] Park SY, Shrestha S, Youn Y-J, Kim J-K, Kim S-Y, Kim HJ, Park S-H, Ahn W-G, Kim S, Lee MG, Jung K-S, Park YB, Mo E-K, Ko Y, Lee S-Y, Koh Y, Park MJ, Song D-K and Hong C-W (2017) Autophagy primes neutrophils for neutrophil extracellular trap formation during sepsis. American Journal of Respiratory and Critical Care Medicine 196, 577–589. 28358992 10.1164/rccm.201603-0596OC

[ref46] Pilsczek FH, Salina D, Poon KK, Fahey C, Yipp BG, Sibley CD, Robbins SM, Green FH, Surette MG and Sugai M (2010) A novel mechanism of rapid nuclear neutrophil extracellular trap formation in response to *Staphylococcus aureus*. The Journal of Immunology 185, 7413–7425. 21098229 10.4049/jimmunol.1000675

[ref47] Pols JW (1960) Studies on bovine besnoitiosis with special reference to the aetiology. Onderstepoort Journal of Veterinary Research 28, 265–356.

[ref48] Reichel M, Muñoz-Caro T, Sanchez Contreras G, Rubio García A, Magdowski G, Gärtner U, Taubert A and Hermosilla C (2015) Harbour seal (*Phoca vitulina*) PMN and monocytes release extracellular traps to capture the apicomplexan parasite *Toxoplasma gondii*. Developmental & Comparative Immunology 50, 106–115. 25681075 10.1016/j.dci.2015.02.002

[ref49] Remijsen Q, Berghe TV, Wirawan E, Asselbergh B, Parthoens E, De Rycke R, Noppen S, Delforge M, Willems J and Vandenabeele P (2011a) Neutrophil extracellular trap cell death requires both autophagy and superoxide generation. Cell Research 21, 290–304. 21060338 10.1038/cr.2010.150 PMC3193439

[ref50] Remijsen Q, Kuijpers TW, Wirawan E, Lippens S, Vandenabeele P and Vanden Berghe T (2011b) Dying for a cause: NETosis, mechanisms behind an antimicrobial cell death modality. Cell Death and Differentiation 18, 581–588. 21293492 10.1038/cdd.2011.1 PMC3131909

[ref51] Rinaldi L, Maurelli MP, Musella V, Bosco A, Cortes H and Cringoli G (2013) First cross-sectional serological survey on *Besnoitia Besnoiti* in cattle in Italy. Parasitology Research 112, 1805–1807. 23274487 10.1007/s00436-012-3241-y

[ref52] Rochael NC, Guimaraes-Costa AB, Nascimento MTC, DeSouza-Vieira TS, Oliveira MP, Garcia e Souza LF, Oliveira MF and Saraiva EM (2015) Classical ROS-dependent and early/rapid ROS-independent release of neutrophil extracellular traps triggered by *Leishmania* Parasites. Scientific Reports 5, 18302. 26673780 10.1038/srep18302 PMC4682131

[ref53] Rostaher A, Mueller Ralf S, Majzoub M, Schares G and Gollnick NS (2010) *Bovine besnoitiosis* in Germany. Veterinary Dermatology 21, 329–334. 20230585 10.1111/j.1365-3164.2009.00813.x

[ref54] Schares G, Basso W, Majzoub M, Cortes HCE, Rostaher A, Selmair J, Hermanns W, Conraths FJ and Gollnick NS (2009) First in vitro isolation of *Besnoitia Besnoiti* from chronically infected cattle in Germany. Veterinary Parasitology 163, 315–322. 19477592 10.1016/j.vetpar.2009.04.033

[ref55] Schares G, Basso W, Majzoub M, Rostaher A, Scharr JC, Langenmayer MC, Selmair J, Dubey JP, Cortes HC and Conraths FJ (2010) Comparative evaluation of immunofluorescent antibody and new immunoblot tests for the specific detection of antibodies against *Besnoitia Besnoiti* tachyzoites and bradyzoites in bovine sera. Veterinary parasitology 171, 32–40. 20378250 10.1016/j.vetpar.2010.03.017

[ref56] Schulz KCA (1960) A report on naturally acquired besnoitiosis in bovines with special reference to its pathology. Journal of the South African Veterinary Association 31, 21–36.

[ref57] Sharif S, Jacquiet P, Prevot F, Grisez C, Raymond-Letron I, Semin MO, Geffré A, Trumel C, Franc M, Bouhsira É and Liénard E (2019) Stomoxys calcitrans, mechanical vector of virulent *Besnoitia Besnoiti* from chronically infected cattle to susceptible rabbit. Medical and Veterinary Entomology 33, 247–255. 30666684 10.1111/mve.12356 PMC6850491

[ref58] Silva LMR, Muñoz Caro T, Gerstberger R, Vila-Viçosa MJM, Cortes HCE, Hermosilla C and Taubert A (2014) The apicomplexan parasite *Eimeria Arloingi* induces caprine neutrophil extracellular traps. Parasitology Research 113, 2797–2807. 24849865 10.1007/s00436-014-3939-0

[ref59] Skendros P, Mitroulis I and Ritis K (2018) Autophagy in neutrophils: from granulopoiesis to neutrophil extracellular traps. Frontiers in Cell and Developmental Biology 6, 109. 30234114 10.3389/fcell.2018.00109 PMC6131573

[ref60] Tainchum K, Shukri S, Duvallet G, Etienne L and Jacquiet P (2018) Phenotypic susceptibility to pyrethroids and organophosphate of wild *Stomoxys Calcitrans* (Diptera: muscidae) populations in southwestern France. Parasitology Research 117, 4027–4032. 30324257 10.1007/s00436-018-6109-y

[ref61] Tanida I, Ueno T and Kominami E (2008) LC3 and autophagy. In Deretic V (ed.), Autophagosome and Phagosome. *Methods in Molecular Biology*, 445. Humana Press, pp. 77–88. 10.1007/978-1-59745-157-4_4 18425443

[ref62] Trujillo LML and Benavides BB (2011) Bovine besnoitiosis: present in Colombia? Revista LaSallista de Investigación 8, 154–162.

[ref63] Ullah I, Ritchie ND and Evans TJ (2017) The interrelationship between phagocytosis, autophagy and formation of neutrophil extracellular traps following infection of human neutrophils by *Streptococcus pneumoniae*. Innate Immunity 23, 413–423. 28399692 10.1177/1753425917704299 PMC5505230

[ref64] Villagra-Blanco R, Silva LMR, Muñoz-Caro T, Yang Z, Li J, Gärtner U, Taubert A, Zhang X and Hermosilla C (2017a) Bovine polymorphonuclear neutrophils cast neutrophil extracellular traps against the abortive parasite *Neospora caninum*. Frontiers in Immunology 8, 606. 28611772 10.3389/fimmu.2017.00606 PMC5447047

[ref65] Villagra-Blanco R, Silva LMR, Aguilella-Segura A, Arcenillas-Hernández I, Martínez-Carrasco C, Seipp A, Gärtner U, Ruiz de Ybañez R, Taubert A and Hermosilla C (2017b) Bottlenose dolphins (*Tursiops truncatus*) do also cast neutrophil extracellular traps against the apicomplexan parasite *Neospora caninum*. International Journal for Parasitology: Parasites and Wildlife 6, 287–294. 28951834 10.1016/j.ijppaw.2017.09.002 PMC5607148

[ref66] Villagra-Blanco R, Silva LMR, Conejeros I, Taubert A and Hermosilla C (2019) Pinniped- and cetacean-derived ETosis contributes to combating emerging apicomplexan parasites (*Toxoplasma Gondii*, *Neospora caninum*) circulating in marine environments. Biology 8, 12. 30857289 10.3390/biology8010012 PMC6466332

[ref67] Vogelsang EG and Gallo P (1941) *Globidium Besnoiti* (Marotel, 1912) y habronemosis cutanea en bovinos de Venezuela. Review of Medicine Veterinary Parasitology Caracas 3, 153–155.

[ref68] Weissmann G, Smolen JE and Korchak HM (1980) Release of inflammatory mediators from stimulated neutrophils. New England Journal of Medicine 303, 27–34. 6246431 10.1056/NEJM198007033030109

[ref69] Yipp BG and Kubes P (2013) NETosis: how vital is it? Blood 122, 2784–2794. 24009232 10.1182/blood-2013-04-457671

[ref70] Zhou E, Conejeros I, Velásquez ZD, Muñoz-Caro T, Gärtner U, Hermosilla C and Taubert A (2019) Simultaneous and positively correlated NET formation and autophagy in *Besnoitia Besnoiti* tachyzoite-exposed bovine polymorphonuclear neutrophils. Frontiers in Immunology 10, 1131. 31191523 10.3389/fimmu.2019.01131 PMC6540735

